# Schwann cell-encapsulated chitosan-collagen hydrogel nerve conduit promotes peripheral nerve regeneration in rodent sciatic nerve defect models

**DOI:** 10.1038/s41598-023-39141-2

**Published:** 2023-07-24

**Authors:** Hiroaki Takeya, Shun Itai, Hiroo Kimura, Yuta Kurashina, Tsuyoshi Amemiya, Narihito Nagoshi, Takuji Iwamoto, Kazuki Sato, Shinsuke Shibata, Morio Matsumoto, Hiroaki Onoe, Masaya Nakamura

**Affiliations:** 1 Department of Orthopaedic Surgery, Keio University School of Medicine, 35 Shinanomachi Shinjuku-Ku, Tokyo, 160-8582 Japan; 2 Department of Mechanical Engineering, Faculty of Science and Technology, Keio University, 3-14-1 Hiyoshi, Kohoku-Ku, Yokohama-Shi, Kanagawa, 223-8522 Japan; 3 Division of Medical Science, Graduate School of Biomedical Engineering, Tohoku University, 1-1 Seiryomachi, Aoba-Ku, Sendai, Miyagi 980-8574 Japan; 4 Division of Advanced Mechanical Systems Engineering, Institute of Engineering, Tokyo University of Agriculture and Technology, 2-24-16 Nakacho, Koganei-Shi, Tokyo, 184-8588 Japan; 5 Institute for Integrated Sports Medicine, Keio University School of Medicine, 35 Shinanomachi Shinjuku-Ku, Tokyo, Japan; 6 Division of Microscopic Anatomy, Niigata University Graduate School of Medical and Dental Sciences, 1-757 Asahimachi-Dori, Chuo-Ku, Niigata, 951-8510 Japan

**Keywords:** Regeneration and repair in the nervous system, Experimental models of disease

## Abstract

Chitosan has various tissue regeneration effects. This study was designed to investigate the nerve regeneration effect of Schwann cell (SC)-encapsulated chitosan-collagen hydrogel nerve conduit (CCN) transplanted into a rat model of sciatic nerve defect. We prepared a CCN consisting of an outer layer of chitosan hydrogel and an inner layer of collagen hydrogel to encapsulate the intended cells. Rats with a 10-mm sciatic nerve defect were treated with SCs encapsulated in CCN (CCN+), CCN without SCs (CCN−), SC-encapsulated silicone tube (silicone+), and autologous nerve transplanting (auto). Behavioral and histological analyses indicated that motor functional recovery, axonal regrowth, and myelination of the CCN+ group were superior to those of the CCN− and silicone+ groups. Meanwhile, the CCN− and silicone+ groups showed no significant differences in the recovery of motor function and nerve histological restoration. In conclusion, SC-encapsulated CCN has a synergistic effect on peripheral nerve regeneration, especially axonal regrowth and remyelination of host SCs. In the early phase after transplantation, SC-encapsulated CCNs have a positive effect on recovery. Therefore, using SC-encapsulated CCNs may be a promising approach for massive peripheral nerve defects.

## Introduction

Tensionless nerve repair is a standard suture technique for cases in which the severed peripheral nerves are coaptated^1^. Autologous nerve transplantation is the gold standard for treatment if the two nerve stumps have a large gap and tensionless sutures cannot be achieved^2^. However, autologous nerve transplantation has disadvantages, such as donor site morbidity and prolonged operation time^3–5^. To address these problems, artificial nerve conduits have been recently developed. The materials for artificial nerve conduits should ideally have no adverse effect on nerve regeneration during the process of degradation^6^.

The basic component of chitosan is chitin, a long-chain polymer of *N*-acetylglucosamine obtained from the exoskeletons of arthropods. Chitin is the second most abundant natural polysaccharide after cellulose^7^. The deacetylated form of chitin is chitosan, and it can be produced at a low cost by alkaline hydrolysis of chitin^8^. Chitosan has been an attractive material for wound-healing applications since the 1980s because of its biological properties, including biocompatibility, biodegradability, and low to no toxicity^9^. However, chitosan is a relatively new material in the field of peripheral nerve regeneration^10^. It was previously completely degraded in vivo and did not release toxic metabolites that could potentially harm the nerve regeneration process during degradation^8^. In contrast, chitosan degradation metabolites have been reported to promote axonal regeneration^11,12^. In animal studies, chitosan artificial nerve conduits encouraged nerve regeneration^13,14^. In clinical randomized controlled trials, recovery from peripheral nerve injury at the finger was better with a chitosan artificial nerve conduit than with simple sutures^15^. Reaxon^®^ (Medovent GmbH, Mainz, Germany) was the first chitosan artificial nerve conduit launched in June 2014.

Despite remarkable advancements in artificial nerve conduits, artificial nerve grafting is still clinically recommended for finger nerve defects of up to 30 mm^16^, and autologous nerve transplantation remains the gold standard for treating extensive peripheral nerve defects^17^. Artificial nerve grafting is still inferior to autologous nerve transplantation for massive peripheral nerve defects for several reasons, including the lack of neurotrophic factors, fibrin matrix bridges, and Schwann cells (SCs)^18–20^. To overcome these drawbacks, various studies have attempted to improve the results of artificial nerve grafting. These studies found that hybridization of artificial nerve conduit materials was beneficial for nerve restoration. Chitosan was also reported to promote nerve regeneration treatment for nerve defects when hybridized with collagen, polyglycolic acid, and polylactide acid by utilizing the advantages of these materials^13,21,22^. Artificial nerve conduits act as a delivery system by adding cells such as SCs or growth factors such as nerve growth factor (NGF), glial cell line-derived neurotrophic factor (GDNF), and fibroblast growth factor (FGF), and the released factors from the conduits stimulate nerve regeneration^23–25^. In addition, specific cells such as SCs, mesenchymal stem cells, and induced pluripotent stem cells are encapsulated within artificial nerve conduits to encourage nerve regeneration^14,26,27^.

We previously proposed a technique to produce a double-layered hydrogel collagen tube, that is, a double-layered heterogeneous hydrogel artificial nerve conduit with an outer layer of chitosan hydrogel and an inner layer of collagen hydrogel, that could encapsulate cells to enhance peripheral nerve restoration with cellular support^28^. We applied this technology to fabricate chitosan-collagen hydrogel nerve conduits (CCNs), with an outer layer of chitosan hydrogel and an inner layer of collagen hydrogel, that could encapsulate cells to enhance peripheral nerve restoration with cellular support^29^. The results showed that the CCN encapsulated in SCs induced axonal regrowth in vitro^29^. The present study aimed to assess the efficacy of SC-encapsulated CCNs in peripheral nerve regeneration and functional recovery in vivo.

## Methods

### Ethics declaration

This experiment was approved (approval number 17024) by the Keio University Institutional Animal Care and Use Committee (Tokyo, Japan). The study complied with the ARRIVE guidelines and all the following methods were performed in accordance with the relevant guidelines and regulations.

### Isolation and cultivation of SCs

In total, three 6-week-old Sprague–Dawley (SD) rats (Sankyo Labs, Tokyo, Japan) were used for SC isolation and cultivation, which were performed according to the methods described by Morrisey et al.^30^ and Meijs et al.^31^. Briefly, the rats were deeply anesthetized with an intraperitoneal injection of medetomidine hydrochloride (0.375 mg/kg), midazolam (2 mg/kg), and butorphanol (2.5 mg/kg). A dorsal longitudinal skin incision was made in the gluteal area, and the sciatic nerve was exposed by splitting the gluteal muscle. The sciatic nerve was excised and placed in a plastic culture dish containing medium (high glucose Dulbecco’s Modified Eagle Medium [DMEM] [Nacalai Tesque, Kyoto, Japan], supplemented with 10% fetal bovine serum [FBS] and 0.5% penicillin and streptomycin), and the epineurium was carefully removed using microforceps. The explants were transferred to a new plastic culture dish containing the medium and incubated at 37 °C under 5% CO_2_.

Pre-degeneration was performed on the culture dish; the medium was changed every 2–3 days, and the culture dish was changed once a week. After 2 weeks, the explants were cut into 1-mm slices and digested with Accutase^®^ (Nacalai Tesque, Kyoto, Japan) at 37 °C for 60 min. The digested explants were added with horse serum and minced using a pipette. The solution containing the cells was centrifuged at 1000 rpm for 5 min, and the supernatants were discarded. The cell pellets were resuspended in culture medium (DMEM supplemented with 10% FBS, 2 µmol/l forskolin [Sigma, MO, USA], 50 ng/ml FGF-2 [ReproTech, MO, USA], 2 nmol/l heregulin [ReproTech, MO, USA], and 0.5% penicillin and streptomycin). The cells were seeded in plastic culture dishes coated with poly-lysin and incubated at 37 °C under 5% CO_2_. The culture medium was changed every 2–3 days, and the cultured cells were passaged to confluence.

To analyze the survival of transplanted cells in vivo, the cultured cells were infected with lentivirus to express *ff*Luc^32^, a fusion protein of a green fluorescence protein (modified from Venus) and luciferase 2, on day 2 at P2. The cells were expanded until three passages and used for the subsequent experiments.

### Immunofluorescence

The characteristics of the cultured SCs were evaluated using immunocytochemistry. The cells were transferred to poly-lysin-coated 8-well plastic-bottomed glass chambers at passage 3 and cultured in the culture medium for 7 days. The cells were then fixed with 4% paraformaldehyde (PFA), washed with phosphate-buffered saline (PBS, 0.1 M), and blocked with blocking buffer (Nacalai Tesque, Kyoto, Japan) for 60 min at room temperature. The cells were then washed with 0.1 M PBS and incubated overnight at 4 °C with a solution containing S-100 (Dako Denmark A/S, Glostrup, Denmark), Thy-1 (Novus Biologicals, CO, USA), and SOX-10 (Proteintech, IL, USA) as primary antibodies. The cells were rinsed with 0.1 M PBS and incubated with the secondary antibodies for 1 h at room temperature. The cells were observed under a fluorescence microscope (BZ9000, Keyence, Osaka, Japan).

### Preparation of SC-containing nerve conduits

**Figure 1:**
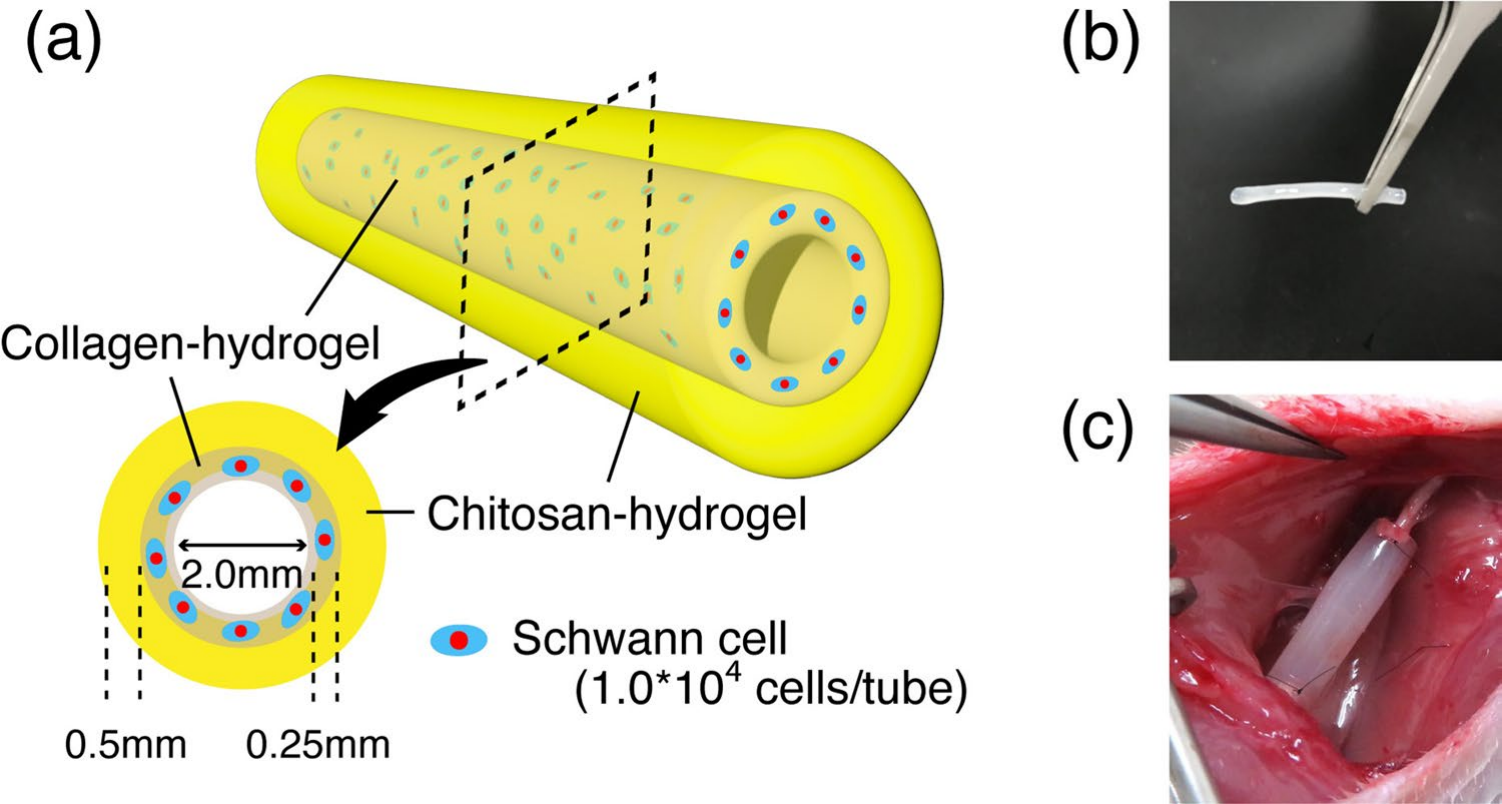
Characteristics of CCN. (**a**) The concept of our chitosan-collagen hydrogel nerve conduit (CCN). In this study, the inner lumen is 2.0 mm in diameter, and the thickness of the chitosan and collagen layers are 0.5 mm and 0.25 mm, respectively. Schwann cells are encapsulated in the inner layer at 1.0 × 10^4^ cells/tube. (**b**) An image of the CCN before transplantation. (**c**) An image of the CCN after transplantation to a sciatic nerve.

The CCNs used in this study had an outer layer of chitosan hydrogel (inner diameter: 2.5 mm; outer diameter: 3.5 mm) and an inner layer of collagen hydrogel (inner diameter: 2.0 mm; outer diameter: 2.5 mm) (Fig. 1a,b). CCNs were fabricated as previously described^28,29^. The conduit was produced using two simple molding steps. The chitosan layer was fabricated using a mold of two glass capillaries of different diameters (outer glass capillary: hollow glass tube 3.5-mm inner diameter; inner glass capillary: solid glass rod 2.5 mm in diameter). Two glass capillaries for the chitosan layer were assembled to serve as molds, and the chitosan pre-gel acid solution was injected into the inner space of the mold using a syringe. The mold was then placed in a sodium hydroxide solution to neutralize and solidify the chitosan. After 4 days, the mold was removed to collect the chitosan hydrogel tube.

A collagen layer was fabricated in chitosan hydrogel tubes using a similar procedure. The chitosan hydrogel tube was placed into a hollow glass tube as the outer glass capillary, and a 2-mm diameter glass rod was assembled as an inner glass capillary for the collagen layer inside. Cell-suspended collagen (Koken, Tokyo, Japan)-alginate pre-gel solution (Wako, Osaka, Japan), adjusted to a concentration of approximately 5.0 × 10^5^ cells/ml, was injected into the inner space of the mold using a syringe. Thereafter, the mold was incubated at 37 °C to solidify the collagen solution. Finally, the glass capillary was removed to obtain the CCN with approximately 1.0 × 10^4^ cells. The silicone tubes (Tigers Polymer Corporation, Osaka, Japan: 2 mm inner diameter, 3 mm outer diameter) used in this study were sterilized by autoclaving at 121 °C for 20 min. The length of both the nerve conduit and silicone tube was 12 mm, and each of them was filled with 1.0 × 10^4^ cultured SCs.

### Surgical procedure

Forty 6-week-old male SD rats were used for transplantation. The left sciatic nerve was exposed in the same manner as above. The sciatic nerve was resected at the middle of the thigh, and its gap was repaired by fixing the nerve stumps 1 mm inside the end of a tube using a horizontal mattress suture of 9–0 monofilament nylon at each end, leaving a 10-mm interstump gap. The rats were assigned to four groups: (1) CCN with SCs (CCN+ group, n = 10 rats); (2) CCN without SCs (CCN− group, n = 10 rats); (3) silicone tube with SCs (silicone+ group, n = 10 rats), and (4) autograft (auto group, n = 10 rats). In the auto group, a 10-mm gap was reconstructed by turning the resected nerve over and bridging the resected nerve. All procedures were performed under a surgical microscope (Fig. 1c). For tracking the transplanted SCs in vivo, the luciferase substrate d-luciferin (Sumisho Pharma International Corporation, Tokyo, Japan) was injected intraperitoneally in the rats of CCN+ and silicone groups (0.3 mg/g body weight). An in vivo imaging system (IVIS) spectrum and CCD optical macroscopic imaging system (Caliper Life Sciences, MA, USA) were used to measure the emission spectrum of the transplanted cells in both groups.

### Walking track analysis

Walking track analysis was performed every 2 weeks postoperatively to evaluate restoration of motor function of the sciatic nerve in all 40 rats. Postoperatively, the rats were placed on the treadmill instrument, and their footprints were scanned using the DigiGait System (Mouse Specifics, MA, USA). The sciatic functional index (SFI) was calculated using the following formula^33^: SFI = 118.9 ([ETS − NTS]/NTS) − 51.2 ([EPL − NPL]/NPL) − 7.5 (ETS: experimental toe spread, NTS: normal toe spread, EPL: experimental print length, NPL: normal print length).

### Immunofluorescence

At 12 weeks post-transplantation, at the very center of regenerated sciatic nerves between the proximal and distal stitch sites from seven rats in each of the four groups were used for fluorescence immunohistochemistry. The tissues were stained with P0 to evaluate the myelination of the regenerated nerves and with neurofilament heavy (NFH) to evaluate the axons of the regenerated nerves. The regenerated sciatic nerves were then resected and fixed overnight in 4% PFA diluted in 0.1 M PBS. Then, the nerves were dehydrated with 10% sucrose overnight the first night and 30% sucrose overnight the next night. The silicone tubes were cut and removed before fixation with 4% PFA. The fixed regenerated nerve specimens were embedded and frozen in the Tissue-Tek optimal cutting temperature compound (Sakura Finetech Co., Tokyo, Japan). Frozen specimens were cut axially to a thickness of 10 μm using a CM3050 cryostat S (Leica Microsystems GmbH, Wetzlar, Germany). Slides were rinsed with 0.1 M PBS to remove the compounds, dried for 30 min, and then blocked with blocking buffer for 1 h at room temperature. The tissues were incubated overnight at 4 °C in a solution containing P0 as the primary antibody. The tissues were rinsed with 0.1 M PBS and incubated with the secondary antibodies for 1 h at room temperature. The samples were observed under a fluorescence microscope, BZ9000 (Keyence, Osaka, Japan). Fields containing regenerating axons were automatically analyzed using ImageJ (National Institutes of Health) to determine the number of regenerated axons, P0-positive areas, and NFH-positive areas.

### Toluidine blue stain and electron microscopic analysis

Twelve weeks post-transplantation, the central portions of the regenerated nerve from the remaining rats not selected for immunostaining were used for toluidine blue stain and electron microscopic (EM) observation, as described previously^34^. Three rats from each of the four groups were included. Briefly, the tissues were sectioned and fixed in 2.5% glutaraldehyde in 0.1 M phosphate buffer (PB) (pH 7.4) at 4 °C for 24 h. After fixation in 1% OsO_4_ for 2 h, the tissues were dehydrated stepwise with ethanol, acetone, and n-butyl glycidyl ether (QY-1). Thereafter, the Epon concentration was increased stepwise with QY-1, and finally to Epon 100%. After 72 h at 60 °C to enhance the polymerization with pure Epon-embedded tissues, semi-thin sections (1-μm thickness) were stained with 0.1% toluidine blue for 7 min and imaged with BZ9000 (Keyence, Osaka, Japan). The total axon area was calculated semi-automatically using toluidine blue staining with the BZ-9000 analysis software.

Ultrathin sections (70-nm thickness) of the axial regenerated sciatic nerve from the same sample for toluidine blue stain were prepared on copper grids and silicon wafers using an ultramicrotome (Leica UC7, Leica Microsystems GmbH). The sections were then stained with uranyl acetate and lead citrate for 10 min and then observed under transmission electron microscopy (JEM-1400Plus, JEOL Ltd., Tokyo, Japan) and scanning electron microscopy (multiSEM505, Zeiss). For quantitative analysis of axonal regrowth and remyelination, the G-ratio, number of myelinated axons, total axon area, inner diameter (ID) and outer diameter (OD) of myelinated axon, and myelin thickness ([OD-ID]/2) of all myelinated axons were semiautomatically calculated using the software program MyelTracer (https://github.com/HarrisonAllen/MyelTracer)^35^. The parameters were measured in randomly selected seven slices of 2000 magnified EM images in each specimen.

### Statistical analysis

Data were presented as the mean ± standard error of the mean. The normality of data distribution was confirmed using the Shapiro–Wilk test. Statistical tests were performed using a one-way analysis of variance method, and Levene’s test was conducted to evaluate homoscedasticity. Variables with uniform and non-uniform variance were analyzed using Tukey’s HSD method and the Games–Howell method, respectively. All statistical analyses were performed using SPSS 28.0 (IBM Corp., Armonk, NY, USA). Values were considered statistically significant at P < 0.05.

## Results

### In vitro characterization and in vivo survival of the cultured SCs

**Figure 2:**
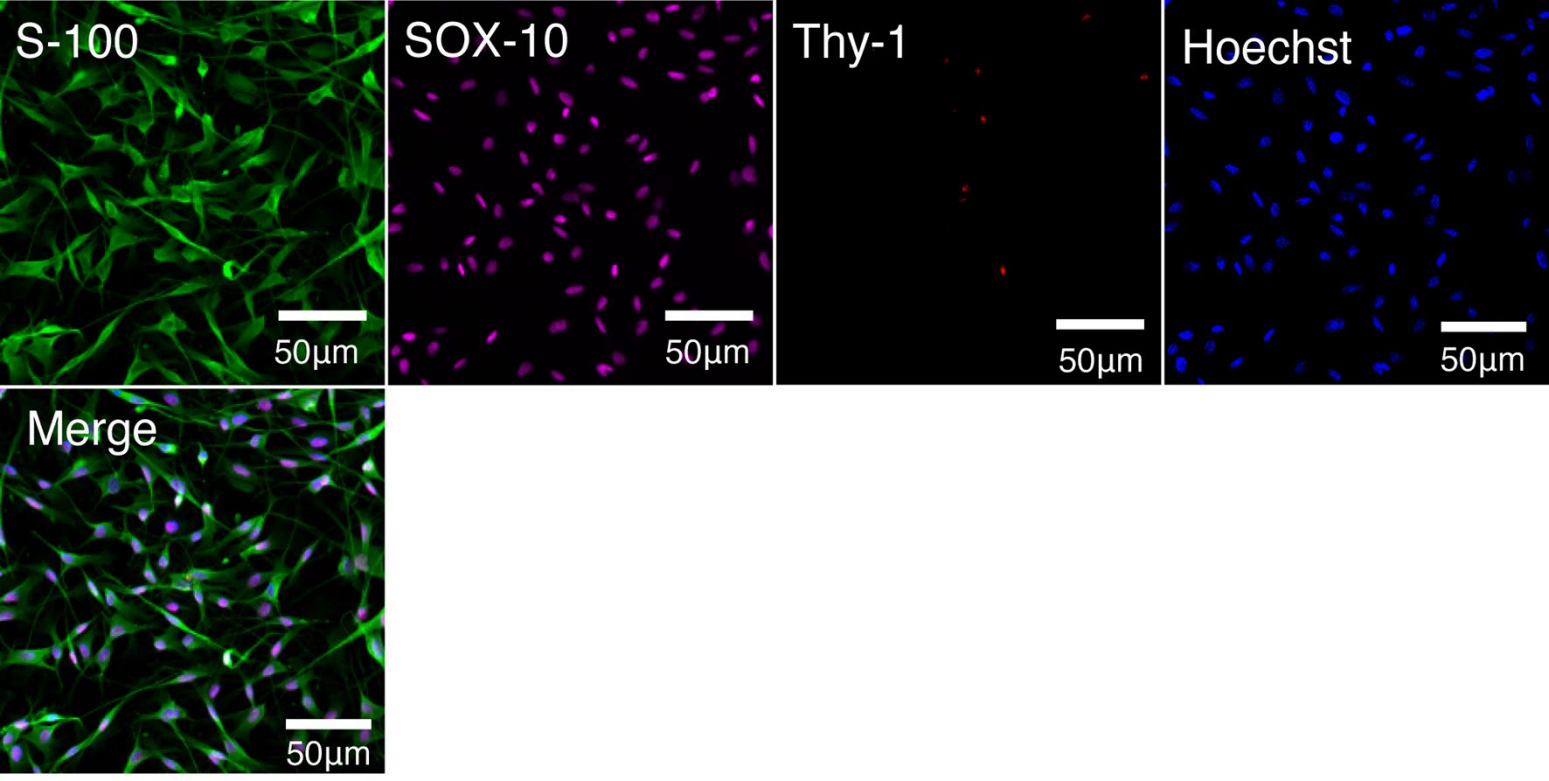
Characteristic of cultured cells. Immunohistochemistry staining of cultured cells for anti-S-100, anti-SOX-10, and anti-Thy-1 antibodies, counterstained with Hoechst. Almost all cultured cells express the Schwann cell markers S-100 and SOX-10 but do not express the fibroblast marker Thy-1.

**Figure 3:**
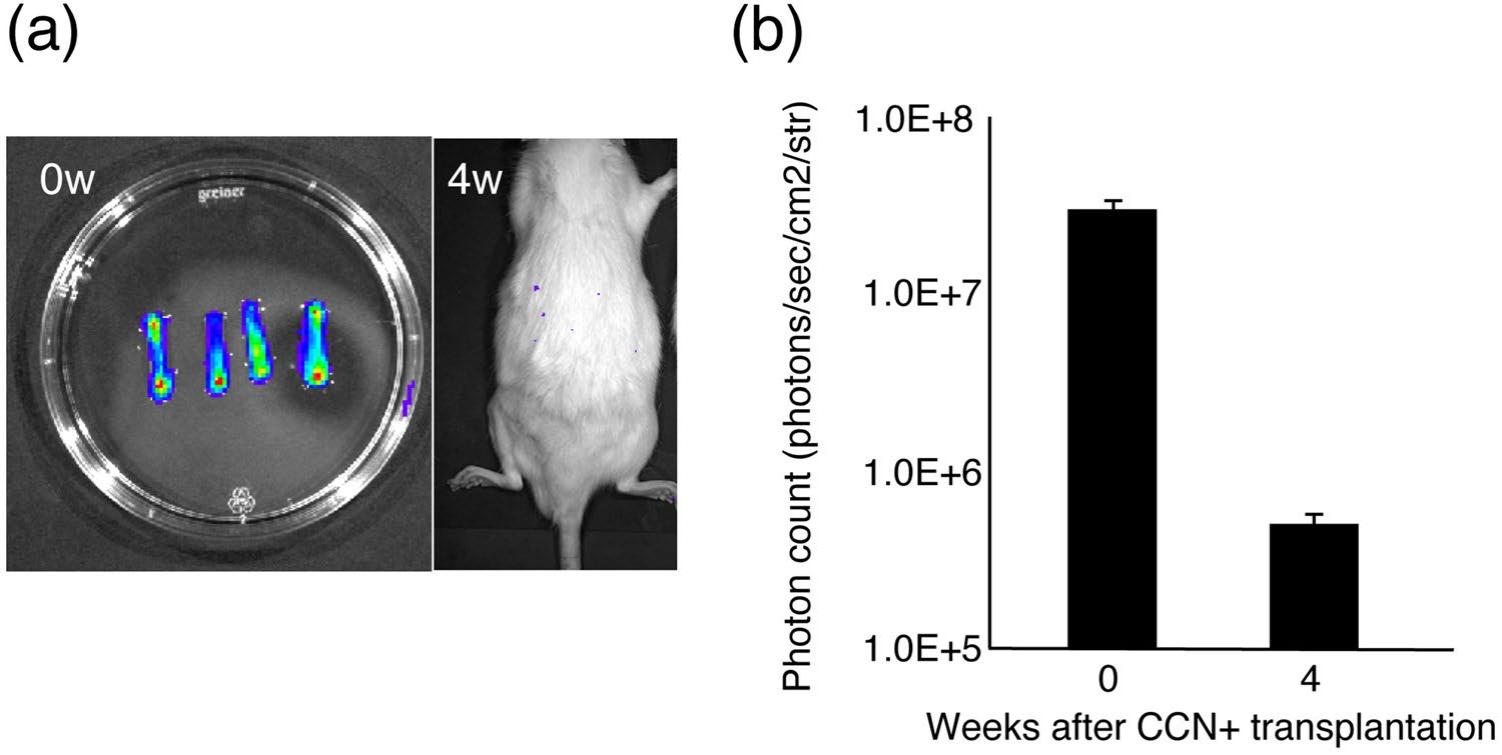
Tracking bioluminescence of transplanted cells. (**a**) Bioluminescence images of the cultured cell-encapsulated CCN and the rat in the CCN+ group at 4 weeks after transplantation. Luminescence disappears at 4 weeks after transplantation. (**b**) Quantitative analysis of photon count from transplanted cells of the cultured cell-encapsulated CCN before transplantation and the CCN+ group at 4 weeks after transplantation.

The majority of cultured cells were positive for S-100 and SOX-10, and few cells were positive for Thy-1. The results indicated that the cultured cells were SCs (Fig. 2). To examine the survival of the cultured SCs after transplantation, cell tracing was performed in the CCN+ and silicone+ groups using IVIS. Although IVIS showed encapsulated SCs surviving in the CCN immediately after encapsulation, IVIS at 4 weeks after transplantation showed no survival in the CCN+ group (Fig. 3a,b).

### Leg motor functional recovery via CCN with SCs

**Figure 4:**
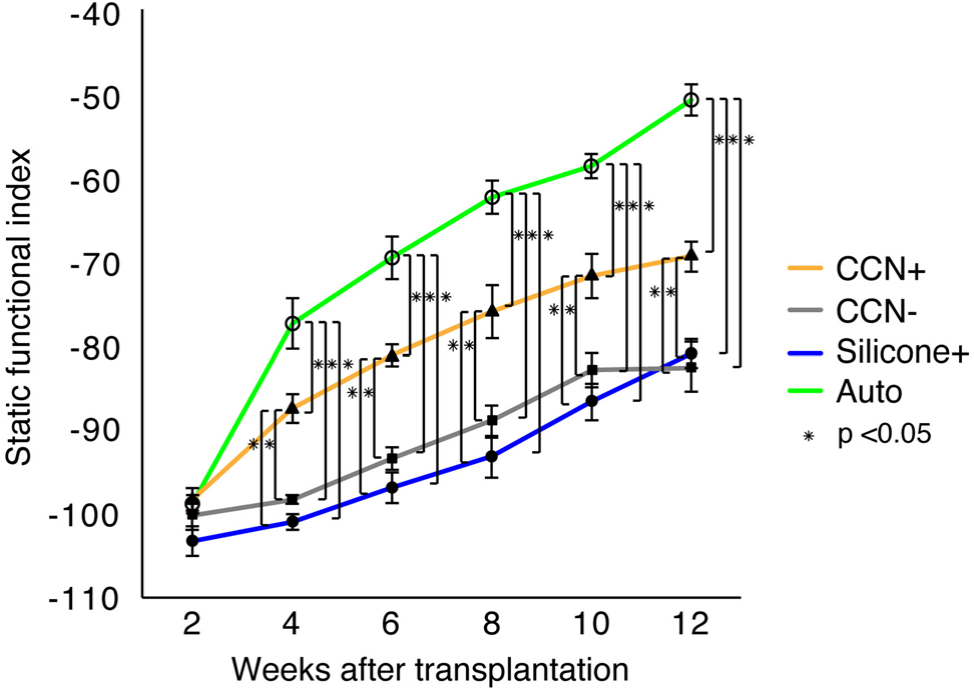
SFI for motor functional evaluation. Walking track analysis shows that the SFI values in the CCN+ group are significantly higher than those in the CCN− and silicone+ groups at 12 weeks after transplantation. However, the differences in the CCN− and silicone+ groups were not statistically significant. These results indicate that CCN promotes motor functional recovery, which is further enhanced by SC encapsulation.

Motor functional restoration was evaluated by calculating the SFI using walking tracks. The auto group showed the highest value, while the CCN+ group showed the second highest from 4 to 12 weeks post-transplantation. The mean SFI value at 12 weeks after transplantation in the auto group (− 50.5 ± 1.9) was significantly higher than that in all other groups (CCN+ group: − 69.1 ± 1.7, P < 0.01; CCN− group: − 82.4 ± 3.0, P < 0.01; silicone+ group: − 80.8 ± 1.7, P < 0.01). The mean SFI value in the CCN+ group was significantly higher than that in the CCN− (P < 0.01) and silicone+ (P < 0.01) groups. The mean SFI value in the CCN− group was not significantly different from that in the silicone+ group (P = 0.95) (Fig. 4).

### Promotion of nerve fiber regeneration by CCN with SCs

**Figure 5:**
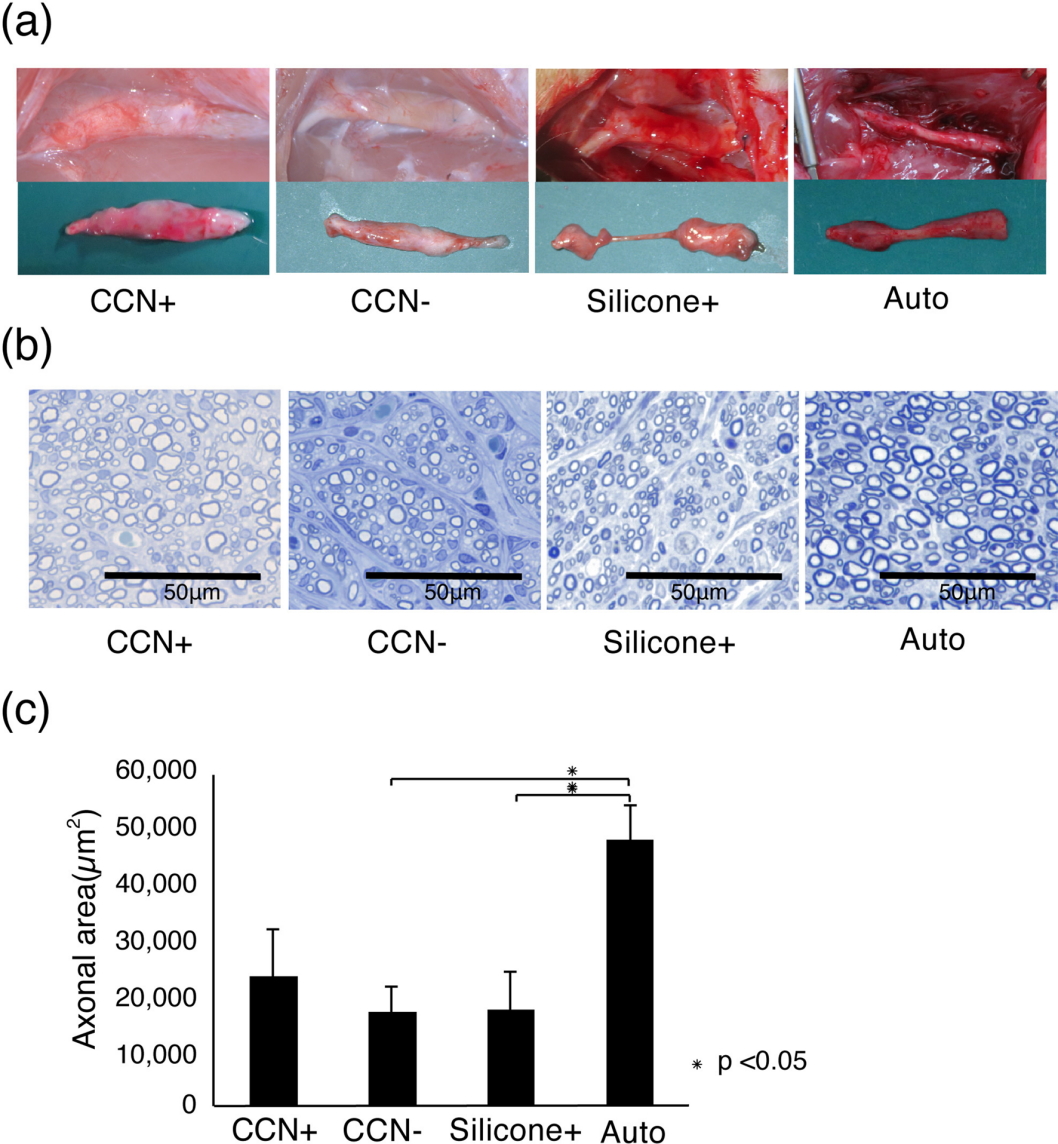
Enhanced nerve regeneration by CCN with SCs. (**a**) Representative images of regenerated sciatic nerves 12 weeks after transplantation. The transplanted CCN in the CCN+ and CCN− groups remains, despite the occurrence of bio-absorption. (**b**) Toluidine blue staining in the central axial sections reveals regenerated nerve fiber in all groups. (**c**) Quantitative analysis of the axonal area of the regenerated nerve fibers (axonal area = cross-sectional area × axon density). The CCN+ group has higher axonal area value than the CCN− and silicone+ groups, but the difference among the three groups is not significant.

The regenerated sciatic nerves were fixed and observed for histological recovery 12 weeks post-transplantation. The transplanted CCNs were covered with fibrous tissue, and the CCN itself remained, whereas 2 out of 10 rats in the silicone+ group showed no nerve regeneration in the tube (Fig. 5a). The toluidine blue staining images of the axial sections demonstrated axonal regeneration in all groups (Fig. 5b). Quantitative analysis of the axonal area of the toluidine blue-stained images revealed that the mean axonal area of the auto group (48,356.5 ± 6267.1 μm^2^) showed significantly greater regrowth of axons (CCN+ group: 23,587.9 ± 8521.9 μm^2^, P = 0.12; CCN− group: 17,070.6 ± 8531.9 μm^2^, P = 0.044; silicone+ group: 17,520.8 ± 6862.3 μm^2^, P = 0.048). The area in the CCN+ group was higher than that in the CCN− (P = 0.90) and silicone+ (P = 0.91) groups, but the difference was not statistically significant (Fig. 5c).

### Effect of SC encapsulation in the CCN on host axonal regrowth and myelination

**Figure 6:**
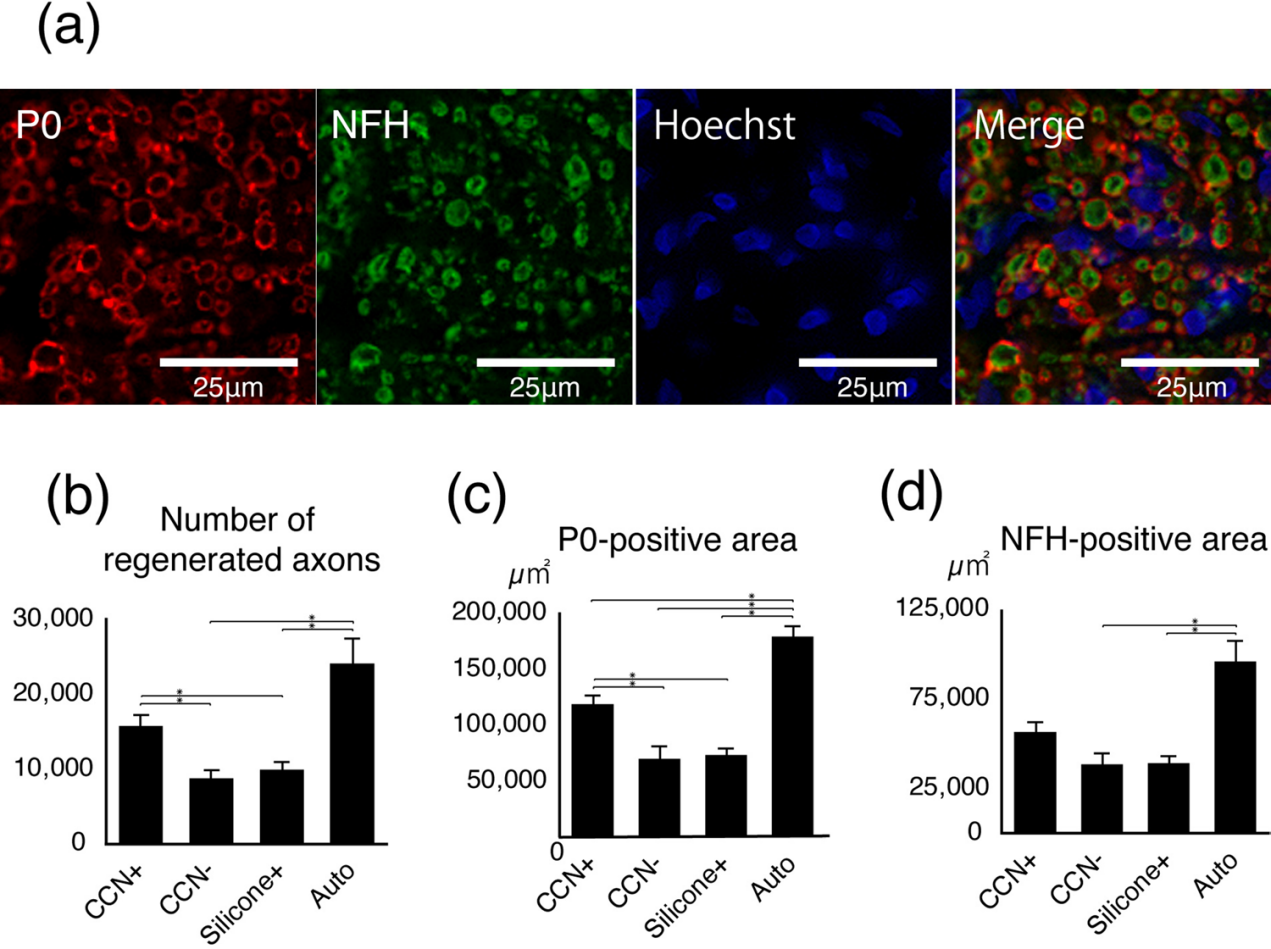
Promoted axonal regrowth and remyelination by CCN with SCs. (**a**) Representative images of immunohistochemistry staining for anti-P0 and anti-NFH antibody, counterstained with Hoechst, at 12 weeks after transplantation. (**b**) Number of regenerated axons, (c) P0-positive area, (**d**) NFH-positive area. *P < 0.05. Quantitative analysis of the axial section immunofluorescence. The number of regenerated axons and P0-positive area in the CCN+ group is significantly larger than those in the CCN− and silicone+ groups, but there is no significant difference between the CCN− and silicone+ groups. These results indicate that SC-encapsulated CCN promotes axonal regrowth and remyelination of regenerated axons.

Fluorescence immunohistochemistry was performed to assess axonal regrowth and myelination in the four groups (Fig. 6a). The number of regenerated axons, P0-positive area, and NFH-positive area were quantitatively evaluated. The auto group had the largest number of regenerated axons (24,143.9 ± 3350.4) (CCN+ group: 15,783.1 ± 1461.4, P = 0.18; CCN− group: 8764.0 ± 1131.6, P = 0.012; silicone+ group: 9939.6 ± 1000.5, P = 0.019). The number of regenerated axons in the CCN+ group was significantly larger than that in the CCN− group (P = 0.013) and silicone+ (P = 0.035) groups. There was no significant difference between the CCN− and silicone+ groups (P = 0.86) (Fig. 6b). The auto group had the largest P0-positive area (179,505.5 ± 9205.0 μm^2^) (CCN+ group: 118,803.7 ± 8195.7 μm^2^, P < 0.01; CCN− group: 70,076.111,270.1 μm^2^, P < 0.01; silicone+ group: 73,628.1 ± 5955.5 μm^2^, P < 0.01). The P0-positive area in the CCN+ group was significantly larger than that in the CCN− group (P < 0.01) and silicone+ (P = 0.018) groups, while there was no significant difference between the CCN− and silicone+ groups (P = 0.99) (Fig. 6c). Similarly, the auto group had the largest NFH-positive area (96,027.5 ± 11,634.4 μm^2^) (CCN+ group: 56,634.8 ± 5621.0 μm^2^, P = 0.45; CCN− group: 38,524.0 ± 6280.4 μm^2^, P = 0.012; silicone+ group: 39,138.9 ± 4053.3 μm^2^, P = 0.031). The NFH-positive area in the CCN+ group was larger than that in the CCN− (P = 0.25) and silicone+ (P = 0.39) groups, but the difference was not statistically significant. There was no significant difference between the CCN− and silicone+ groups (P = 0.99) (Fig. 6d).

**Figure 7:**
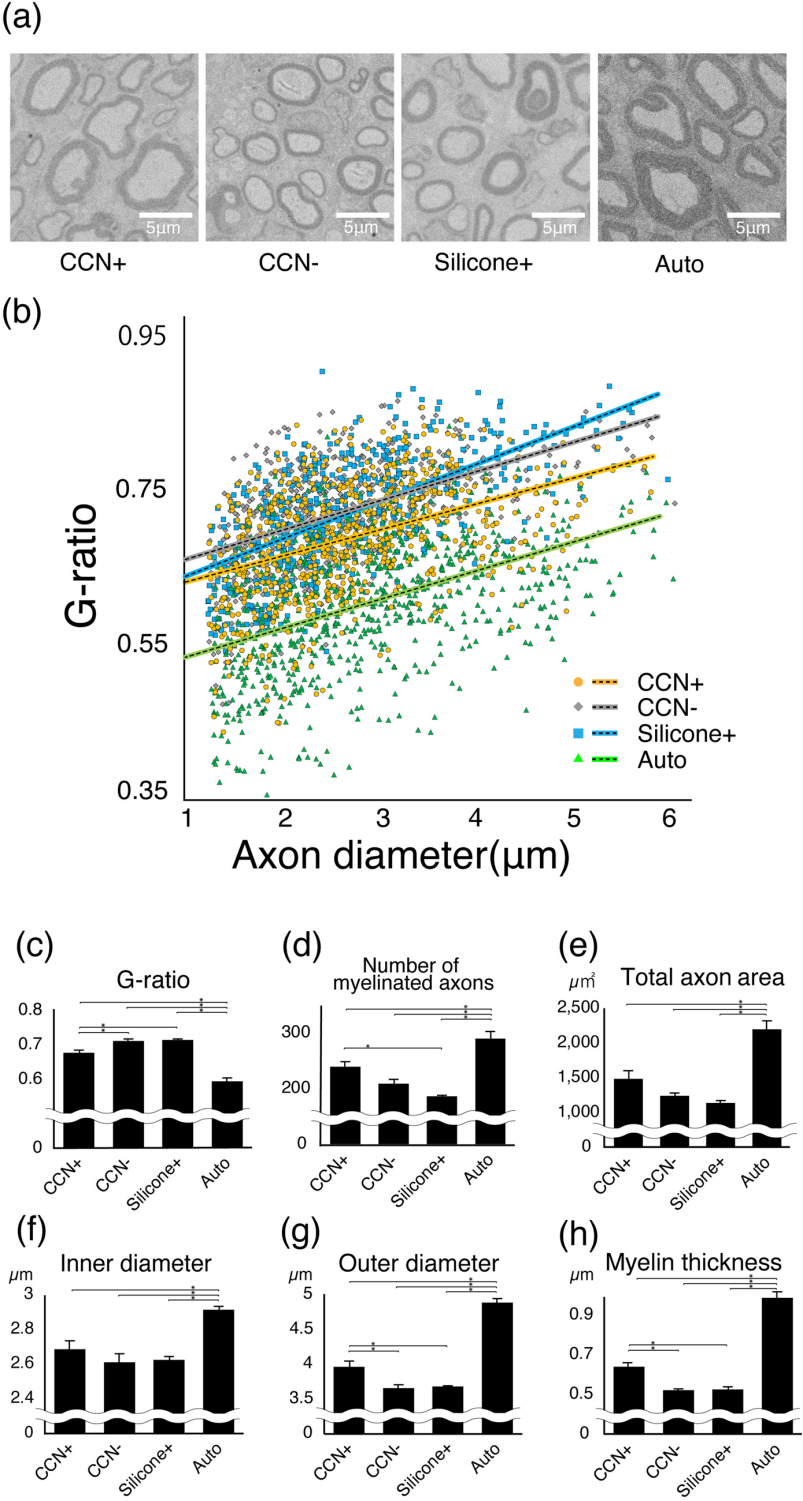
Active myelination detected by electron microscopy. (**a**) Representative electron microscopic images in the central axial sections of all groups at 12 weeks after transplantation. (**b**) Scatterplot of G-ratio and axon diameter in each group. Orange dots: CCN+ group; gray dots: CCN− group; blue dots: silicone+ group; green dots: auto group. Quantitative analysis of the (**c**) G-ratio, (**d**) number of myelinated axons, (**e**) total axon area, (**f**) inner diameter (ID) of myelinated axons, (**g**) outer diameter (OD) of myelinated axons, and (**h**) myelin thickness ([OD-ID]/2). *P < 0.05. Quantitative analysis of the EM in all four groups. The G-ratio, OD of myelinated axons, and myelin thickness in the CCN+ group is significantly higher than those in the CCN− and silicone+ groups, but there is no significant difference between the CCN− and silicone+ groups. These results indicate that the myelinated axons in the CCN+ group have thicker myelin than those in the CCN− and silicone+ groups and that SC-encapsulated CCN promotes remyelination of regenerated axons.

For quantitative evaluation, the G-ratio, number of myelinated axons, total axon area, ID and OD of myelinated axon, and myelin thickness was calculated using electron microscopic analysis (Fig. 7). The average G-ratio in the CCN+ group was 0.67 ± 0.049; CCN− group, 0.70 ± 0.051; silicone+ group, 0.71 ± 0.020; and auto group, 0.59 ± 0.025. The myelin lamella was significantly thicker in the CCN+ group than in the CCN− (P = 0.027) and silicone+ (P = 0.018) groups, while there was no significant difference between the CCN− group and the silicone+ group (P = 0.99). The auto group showed the thickest myelination (CCN+ group, P < 0.01; CCN− group, P < 0.01; and silicone+ group, P < 0.01). The average number of myelinated axons in the CCN+ group was 240.3 ± 9.3; CCN− group, 209.7 ± 8.1; silicone+ group, 187.0 ± 1.7; and auto group, 291 ± 12.9 (CCN+ vs. CCN−: P = 0.15; CCN+ vs. silicone+ : P = 0.013; CCN− vs. silicone+: P = 0.34; auto vs. CCN+: P = 0.017; auto vs. CCN−: P < 0.01; auto vs. silicone+ P < 0.01). The total axon area in the CCN+ group was 1491.2 ± 116.1 μm^2^; CCN− group, 1243.9 ± 42.9 μm^2^; silicone+ group, 1141.1 ± 34.1 μm^2^; and auto group, 2200.8 ± 121.7 μm^2^ (CCN+ vs. CCN−: P = 0.27; CCN+ vs. silicone+: P = 0.088; CCN− vs. silicone+: P = 0.84; auto vs. CCN+: P = 0.02; auto vs. CCN−: P < 0.01; auto vs. silicone+: P < 0.01). The average ID in the CCN+ group was 2.7 ± 0.14 μm; CCN− group, 2.6 ± 0.12 μm; silicone+ group, 2.6 ± 0.070 μm; and auto group, 2.9 ± 0.25 μm (CCN+ vs. CCN−: P = 0.53; CCN+ vs. silicone+: P = 0.67; CCN− vs. silicone+: P = 0.99; auto vs. CCN+: P = 0.011; auto vs. CCN−: P = 0.02; auto vs. silicone+ P = 0.03). The OD in the CCN+ group was 4.0 ± 0.36 μm; CCN− group, 3.7 ± 0.10 μm; silicone+ group, 3.7 ± 0.13 μm; and auto group, 4.9 ± 0.037 μm (CCN+ vs. CCN−: P = 0.018; CCN+ vs. silicone+ : P = 0.027; CCN− vs. silicone+: P = 0.99; auto vs. CCN+: P < 0.01; auto vs. CCN−: P < 0.01; auto vs. silicone+: P < 0.01). The myelin thickness in the CCN+ group was 0.64 ± 0.049 μm; CCN− group, 0.52 ± 0.049 μm; silicone+ group, 0.53 ± 0.020 μm; and auto group, 0.99 ± 0.019 μm (CCN+ vs. CCN−: P = 0.016; CCN+ vs. silicone+: P = 0.02; CCN− vs. silicone+: P = 0.99; auto vs. CCN+: P < 0.01; auto vs. CCN−: P < 0.01; auto vs. silicone+: P < 0.01).

## Discussion

The current study shows that SC-encapsulated CCNs better promote motor functional and histological recovery than CCN itself or SC-encapsulated silicone tubes, although autograft remains superior. Histologically, SC-encapsulated CCNs promote axonal regrowth, as indicated by EM analyses and immunofluorescence analysis of the NFH-positive area. Further, EM analyses and immunofluorescence analysis of the P0-positive area showed that remyelination of the regenerated axons is also enhanced in SC-encapsulated CCNs. Although IVIS analysis showed that the grafted SCs did not survive for more than 4 weeks after transplantation, they contributed to peripheral nerve regeneration.

SCs play several essential roles in nerve regeneration. SCs form the nerve regeneration track at the nerve injury site, known as the band of Bungner^36^, and secrete various factors to develop nerve regeneration. In the early stage of peripheral nerve injury, SCs release cytokines, such as interleukin-6 and leukemia inhibitory factor, that attract macrophages to the nerve and induce axonal regeneration in neurons^37,38^. Moreover, SCs release various neurotrophic growth factors, such as GDNF, NGF, brain-derived neurotrophic factor, and neurotrophin-3, that promote axonal elongation and survival of neurons^39^. Additionally, SC-derived exosomes are used for peripheral nerve regeneration in animal studies. Exosomes are nanovesicles 50–100 nm in diameter that have been shown to mediate intercellular communication^40,41^. SC-derived exosomes are mediators that encourage axonal elongation, and direct injection of SC-derived exosomes after rat sciatic nerve crush injury in vivo has been found to stimulate axonal regeneration^42^. Thus, because SCs are critical for nerve regeneration, the combination of an artificial nerve conduit without cellular components and SC transplantation is expected to improve the outcomes of peripheral nerve injury^43^.

Previous studies where SCs were embedded in nerve conduits showed that transplanted SCs promoted remyelination and revascularization through reconstruction of the microvasculature, resulting in motor functional recovery^43–45^. Hypoxic conditions in the tube negatively affect host SC migration and tissue regeneration, limiting the ability of acellular nerve conduits to improve functional damage^46^. However, combination with SC transplantation improves microvessel density in the regenerated nerve tissue, suggesting that the transplanted SC could stimulate the angiogenesis of regenerated nerve tissues and improve the oxygenation of the artificial nerve conduit^44,45^. In our study, encapsulated SCs in the inner collagen layer had short survival, and thus, we assumed that they affected the transected nerve stumps and their surrounding microvasculature only in the early phase.

We previously showed that CCN with SCs attracted axonal elongation from neurons in vitro^29^. In this study, although transplanted SCs did not survive for more than 4 weeks after surgery, they helped promote nerve regeneration, possibly by secreting cytokines, neurotrophic growth factors, and SC-derived exosomes. Our results support that although transplanted SCs cannot survive long, cell transplantation can be beneficial for nerve regeneration and functional recovery. Although we encapsulated SCs cultured from the sciatic nerve, the ideal cell type for transplantation remains controversial. One of the critical problems in clinical applications is the limited supply of SCs for culture and transplantation. To solve this problem, stem cells such as neural stem cells, mesenchymal stem cells, induced pluripotent stem cells, and SCs derived from these stem cells have been investigated in the field of peripheral nerve injury^47^. Further studies are warranted to elucidate the optimal cell source and its differentiation embedded in the CCN to optimize nerve regeneration and safety.

Biocompatibility, bioabsorbability, and flexibility are required for artificial nerve conduits, and several biodegradable materials have been used, including collagen, polyglycolic acid (PGA), polylactic acid (PLA), and polycaprolactone^48^. Here, we focused on chitosan, which has several beneficial characteristics for use as a bioabsorbable artificial nerve conduit. Serum lysozyme completely degrades chitosan in vivo, and the susceptibility of chitosan to degradation by lysozyme is inversely proportional to the degree of de-*N*-acetylation of chitosan^49^. Additionally, unlike PGA and PLA, chitosan has positive effects on nerve regeneration during in vivo degradation and does not have adverse effects (e.g., pH decrease or inflammatory foreign body reaction)^11,12,50^. Chitooligosaccharide (COS), a degradation product of chitosan, promotes axon regeneration by stimulating SC division and inhibiting apoptosis^11,12^. COS also decreases malondialdehyde activity and increases superoxide dismutase activity, which prevents oxidative stress in SCs^51,52^. Similarly, neuropathic pain associated with post-traumatic neuroma can cause peripheral nerve injury. In this regard, covering the proximal stamp of the transected nerve with chitosan reduces the formation of post-traumatic neuroma and further neuropathic pain; therefore, research has focused on chitosan and its ability to prevent post-traumatic neuroma^53^.

However, although chitosan could be a suitable material for artificial nerve conduits, a single monolayer chitosan artificial nerve conduit lacks cellular support within the tubes^54^. To overcome this drawback, we propose a heterogeneous hydrogel artificial nerve conduit with a two-layered structure consisting of an outer layer of chitosan hydrogel and an inner layer of collagen hydrogel, which can encapsulate cells into the inner layer to promote peripheral nerve regeneration with cell supports^29^. We previously reported that a conduit encapsulated in SCs stimulated axon elongation in vitro^29^. In addition to its ability to encapsulate cells, our CCN has many other benefits. First, the ease of the two-step fabrication is a significant advantage. It is essential to graft an appropriately sized artificial nerve conduit for peripheral nerve injuries^46^. Therefore, the easy control of the inner and outer diameters of the CCN by changing the size of the glass rod and glass tube is one of the advantages^29^. In addition, hollow chitosan tubes can be prepared beforehand because of the two-step fabrication, and transplanted cells can be encapsulated in the inner layer just before surgery^29^. This easy and rapid fabrication allows cell-containing artificial nerve transplantation for clinical applications^29^.

Moreover, our CCN can adjust the degeneration rate^29^. Although the optimal degeneration rate of an artificial nerve conduit is not yet fully understood^55^, the time required for nerve regeneration depends on the location and length of the nerve defect^56^. Controlling the deacetylation of chitosan, concentration of the chitosan solution, and size of the CCN would allow the CCN degeneration rate to be adjusted for each case^29^. In this study, we grafted CCN into rat models of sciatic nerve defects, and at 3 months postoperatively, the residual chitosan layer was still observed outside the regenerated nerve fibers in all CCN+ and CCN− samples. The CCN+ group showed superior histological and functional recovery to the silicone+ group, supporting that SC-encapsulated CCNs could be a feasible hybrid nerve conduit. Meanwhile, nerve regeneration was not significantly different between the CCN− and silicone+ groups. This result implies the need for cell encapsulation for CCN, although various cells aside from SC should be assessed and compared. Therefore, further investigation is warranted to elucidate the optimal cells for transplantation and the effectiveness of CCN itself.

This study has some limitations. First, the sample size was small for complete histological analyses. Second, histological assessment was evaluated only on axial sections at the center of the nerve bridged site similarly to the past studies^14,26,27,46,57,58^. Assessment of axial sections in the different levels (proximal and distal) or sagittal sections would be more valuable, though we simplified the section site for mitigating technical errors and standardizing sample quality. Moreover, this study did not totally confirm SC infection with *ff*Luc, which could be tracked after transplantation using IVIS. Therefore, there is a possibility that some SCs that were not infected with *ff*Luc survived longer than 4 weeks after transplantation, and the exact duration by which SC-encapsulated CCNs survived in vivo remains unclear. Finally, we used the outbred SD rats for transplantation in this study because of their accessibility and cost. Past studies have shown that the majority of SCs transplanted in SD rats remained viable for a few weeks^45^, and even allogeneic SC transplantation promoted peripheral nerve regeneration^59^. However, the possibility of an existing immune reaction that prevents survival and proliferation of the transplanted SCs could not be ruled out. If Lewis rats were used instead of SD rats or immunosuppressants were administered, the transplanted cells could survive longer, and their effectiveness might be increased^60,61^.

In conclusion, transplantation of SC-encapsulated CCNs improved histological and motor functional recovery in a rat sciatic nerve defect model. SC-encapsulated CCNs exert a synergistic effect on peripheral nerve regeneration, especially on axonal regrowth and remyelination of host SCs. In the early phase after transplantation, SCs encapsulated in the inner layer of CCN have a positive impact on functional recovery. Therefore, using SC-encapsulated CCNs may be a promising approach for massive peripheral nerve defects.

## Data Availability

All data analyzed during this study are available from the corresponding author on reasonable request.
